# Influence of prediabetes on the prognosis of patients with myocardial infarction: a meta-analysis

**DOI:** 10.1186/s13098-024-01381-1

**Published:** 2024-07-12

**Authors:** Mengya Zeng, Eyu Sun, Li Zhu, Lingzhi Deng

**Affiliations:** 1 Department of Cardiovascular disease, The Second Affiliated Hospital of Hainan Medical University, Haikou, 570216 China; 2 Directly Affiliated Government Kindergartens of Chenzhou, Chenzhou, 423000 China; 3 Department of Cardiovascular Medicine, The Affiliated Chenzhou Hospital, University of South China, Chenzhou, 423000 China; 4 Department of Cardiovascular Medicine, Chenzhou First People’s Hospital of Hunan Province, No. 102, Luojiajing, Beihu District, Chenzhou, Hunan Province 423000 China

**Keywords:** Acute myocardial infarction, Prediabetes, Major adverse cardiovascular events, Risk factor, Meta-analysis

## Abstract

**Background:**

Previous studies evaluating the association between prediabetes the prognosis of patients with acute myocardial infarction (AMI) showed inconsistent results. The aim of the meta-analysis was to compare the long-term incidence of major adverse cardiovascular events (MACEs) between AMI patients with prediabetes and normoglycemia.

**Methods:**

Relevant prospective cohort studies were obtained by searching Medline, Web of Science, and Embase databases. Only studies with follow-up duration of at least one year were included. A random-effects model was utilized to pool the results by incorporating the influence of heterogeneity.

**Results:**

Twelve studies with 6972 patients with AMI were included. Among them, 2998 were with prediabetes and 3974 were with normoglycemia. During a mean follow-up of 52.6 months, 2100 patients developed MACEs. Compared to those with normoglycemia, AMI patients with prediabetes were associated with a higher incidence of MACEs (risk ratio [RR]: 1.30, 95% confidence interval: 1.07 to 1.58, *p* = 0.008; I^2^ = 67%). Subgroup analysis showed a stronger association between prediabetes and MACEs in studies of patients with mean age ≥ 60 years compared to < 60 years (RR: 1.66 versus 1.10, p for subgroup difference = 0.04), with proportion of men < 75% compared to ≥ 75% (RR: 1.87 versus 1.08, p for subgroup difference = 0.01), and in prediabetes evaluated at or after discharge compared to that evaluated within three days of AMI onset (RR: 1.39 versus 0.78, p for subgroup difference = 0.01).

**Conclusions:**

Prediabetes may be associated with a higher risk of MACEs in patients with AMI.

**Supplementary Information:**

The online version contains supplementary material available at 10.1186/s13098-024-01381-1.

## Introduction

Acute myocardial infarction (AMI) remains a leading cause of mortality and morbidity worldwide, posing significant challenges to public health systems and healthcare providers [1, 2]. Although timely revascularization therapy such as percutaneous coronary intervention (PCI) has reduced the acute mortality of patients with AMI [3], these patients still have increased risk of heart failure (HF) and poor cardiovascular prognosis [4, 5]. Over the past few decades, substantial efforts have been directed towards understanding the intricate interplay between metabolic abnormalities and cardiovascular diseases, including the impact of glycemic status on the prognosis of patients with AMI [6, 7].

Prediabetes, a state characterized by impaired glucose metabolism below the threshold for diabetes diagnosis, has emerged as a crucial intermediary in the spectrum of glucose dysregulation [8, 9]. Clinically, prediabetes refers to status of impaired glucose regulation before the diagnosis of diabetes, which includes impaired fasting glucose (IFG), impaired glucose tolerance (IGT), and mildly elevated glycolated hemoglobin (HbA1c: 5.7 to 6.4%) [10]. Similar to diabetes, people with prediabetes have also been associated with an increased risk of cardiovascular diseases as indicated in a previous meta-analysis [11]. While its association with the risk of developing type 2 diabetes mellitus is well-established [12], the influence of prediabetes on the clinical outcomes of individuals experiencing AMI remains a subject of debate and investigation. Previous studies investigating this association have yielded conflicting results, with some suggesting a detrimental effect of prediabetes on the long-term prognosis of AMI patients [13–17], while others have failed to demonstrate a significant correlation [18–21]. Such inconsistencies may stem from variations in study design, patient characteristics, follow-up duration, and geographic differences among the investigated populations.

In light of these discrepancies, a comprehensive evaluation through a meta-analysis becomes imperative to elucidate the true magnitude of the impact of prediabetes on the incidence of major adverse cardiovascular events (MACEs) following AMI. By synthesizing data from existing prospective cohort studies, this meta-analysis aims to provide a robust assessment of the association between prediabetes and long-term cardiovascular outcomes in patients with AMI.

## Methods

The Preferred Reporting Items for Systematic Reviews and Meta-Analyses (PRISMA 2020) [22, 23] and the Cochrane Handbook for Systematic Reviews and Meta-analyses [24] were followed in this meta-analysis during study design, data collection, statistical analysis, and results interpretation.

### Literature search

To identify studies relevant to the aim of the meta-analysis, we searched Medline, Web of Science, and Embase utilizing the combination of comprehensive search terms involving (“prediabetes” OR “pre-diabetes” OR “prediabetic” OR “pre-diabetic” OR “prediabetic state” OR “borderline diabetes” OR “impaired fasting glucose” OR “impaired glucose tolerance” OR “IFG” OR “IGT”) AND (“myocardial infarction” OR “STEMI” OR “NSTEMI” OR “AMI”) AND (“prognosis” OR “mortality” OR “death” OR “major adverse cardiovascular events” OR “MACE” OR “cohort” OR “prospective” OR “prospectively” OR “risk” OR “incidence” OR “followed” OR “follow-up” OR “longitudinal”). The search was limited to studies in humans. We only considered studies published as full-length articles in peer-reviewed journals in English. As a supplementation, the references of related original and review articles were also manually screened for potentially related studies. The literatures published from the inception of the databases to February 8, 2024 were screened.

### Inclusion and exclusion criteria

The inclusion criteria for the potential studies were: (1) prospective cohort studies published as full-length articles; (2) included patients with AMI, with no limitations of treatments; (3) prediabetes was evaluated at baseline, which was diagnosed according to the methods and diagnostic criteria used in the original studies; (4) patients with AMI were followed for at least one year; and (5) reported the incidence of MACEs, which was compared between patients with prediabetes and normoglycemia at baseline. The definition of MACEs were also consistent with that used among the included studies, which generally includes composite outcome of cardiovascular deaths, non-fatal MI, non-fatal stroke, HF, and repeated PCI.

Exclusion criteria were: (1) cross-sectional studies or retrospective studies, studies including non-AMI patients, studies without the outcome of MACEs; (2) studied did not evaluate prediabetes at baseline; or (3) preclinical studies, reviews, or editorials. If studies with overlapping population were retrieved, the one with the largest sample size was included for the meta-analysis.

### Study quality evaluation and data extraction

The processes of literature search, study identification, study quality evaluation, and data collection were independently conducted by two authors. If disagreement occurred, a consultation with the corresponding author was indicated to resolve the disagreement. We used the Newcastle-Ottawa Scale (NOS) [25] for the assessment of the quality of the included studies. This scale consisted of three aspects, including selection of the population, control of confounders, and outcome measurement and analysis. The total scores of NOS were 1 to 9, with 9 indicating the best quality. The following data was extracted from each study for subsequent analysis, including study information (author, year, and country), participant characteristics (diagnosis, sample size, age, and sex), diagnosis of prediabetes (definition, timing of evaluation, and number of participants with prediabetes), outcome information (follow-up durations and number of patients who developed MACEs), and variables adjusted when the association between prediabetes and MACEs in patients with AMI was reported.

### Statistics

The association between prediabetes and long-term incidence of MACEs after AMI was summarized as risk ratio (RR) and corresponding 95% confidence interval (CI). For studies reporting only RR of univariate analysis, these data was extracted; for studies reporting adjusted RR from multivariate analyses, RRs from the most adequately adjusted model were extracted. By using 95% CIs or p-values, RRs and standard errors (SEs) could be calculated, and a subsequent logarithmical transformation kept the variance stabilized and normalized. We combined the log RR or log hazard ratios (HR) and corresponding standard errors by the inverse variance approach. Cochrane Q test and I^2^ statistics were used to estimate study heterogeneity [26], and the significantly statistical heterogeneity is reflected by an I^2^ > 50%. The results were combined using a random-effects model incorporating heterogeneity’s influence [24]. The sensitivity analyses by omitting one study at a time (leave-one-out test) were performed to investigate the robustness of the findings. The predefined subgroup analyses were also performed to evaluate the influences of study characteristics on the outcome. The medians of the continuous variables were used as the cutoffs for defining subgroups. In addition, a univariate meta-regression analysis was also performed to investigate the potential influence of study characteristics in continuous variables on the association between prediabetes and long-term incidence of MACEs after AMI [24]. The estimation of publication bias underlying the meta-analysis was firstly achieved by construction of the funnel plots and visual inspection of the plot symmetry [27]. An Egger’s regression test was also performed [27]. The statistical analysis was carried out using RevMan (Version 5.1; Cochrane Collaboration, Oxford, UK) and Stata software (version 12.0; Stata Corporation, College Station, TX). A two-sided *p* < 0.05 suggests statistical significance.

## Results

### Study inclusion

**Fig. 1:**
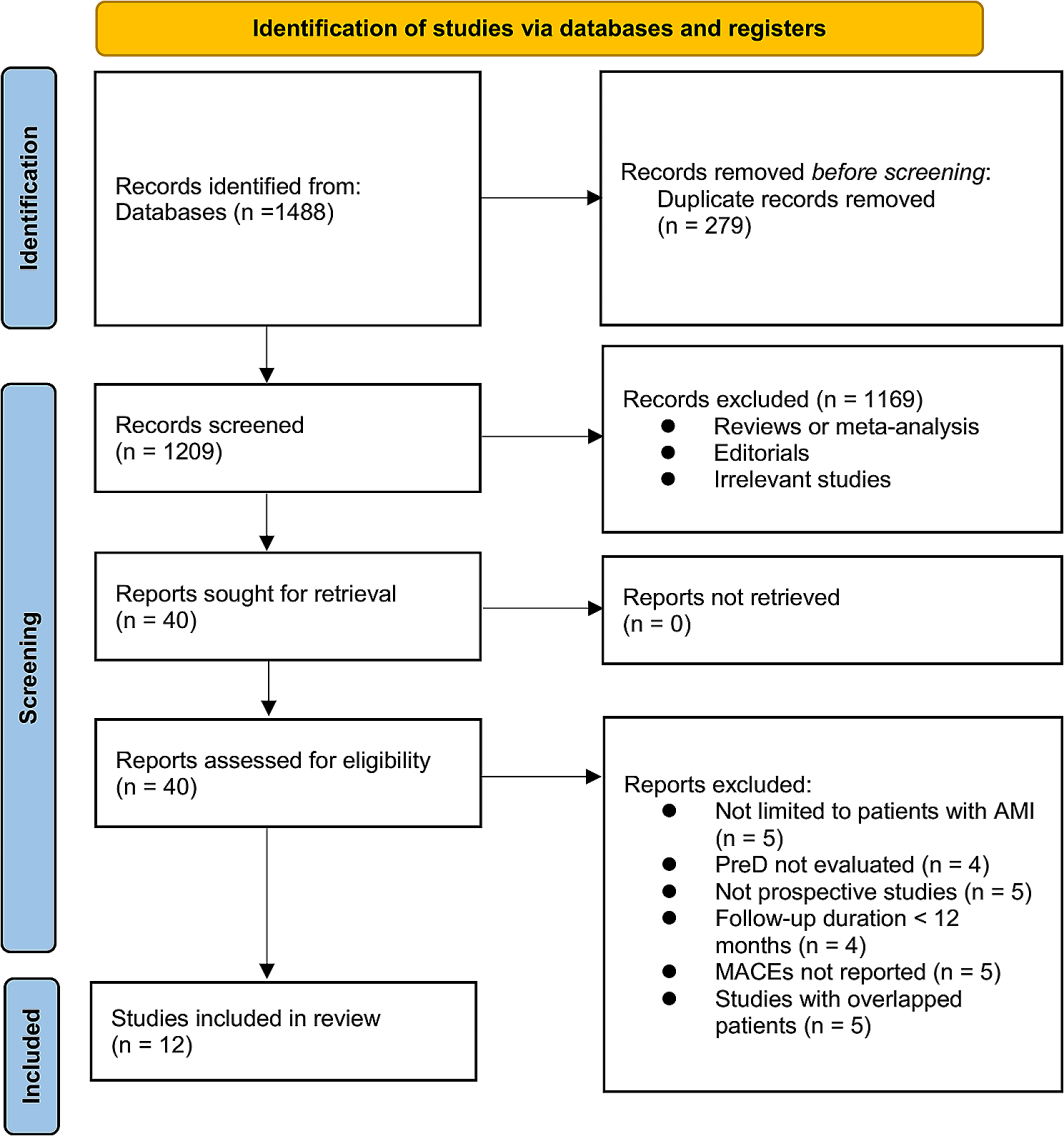
The flowchart depicts the process of database search and study inclusion

The process of study inclusion is presented in Fig. 1. In brief, 1488 potentially relevant records were obtained after comprehensive search of the three databases, and 279 of them were excluded due to the duplication. Subsequently, a screening via titles and abstracts of the remained records further excluded 1169 studies, mostly because they were not related to the aim of the meta-analysis. Accordingly, the full texts of the 40 left records were read by two independent authors, and 28 of them were further removed for the reasons listed in Fig. 1. Finally, twelve observational studies were considered to be suitable for the subsequent quantitative analyses [13–21, 28–30].

### Overview of study characteristics

Table 1 presents the summarized characteristics of the included studies. Overall, twelve prospective studies with 6972 patients with AMI were included [13–21, 28–30]. These studies were published between 2009 and 2021, and conducted in Denmark, Sweden, Norway, the United States, Poland, Japan, Sweden, Russia, Italy, and China. The mean ages of the participants were 54.9 to 67.6 years, and the proportions of men were 54.2 to 84.5%. Among these studies, the diagnosis of prediabetes was based on IFG solely in two studies [13, 18], IGT solely in two studies [15, 16], mildly elevated HbA1c in one study [17], IFG and/or IGT in five studies [15, 19–21, 29], and IFG/IGT/mildly elevated HbA1c in two studies [29, 30]. Prediabetes was evaluated within three days after AMI onset in two studies [19, 21], at or after discharge in seven studies [13–16, 19, 21, 29], while the exact timing for the diagnosis of prediabetes was not reported in another three studies [17, 29, 30]. Accordingly, 2998 of the included patients were with prediabetes and 3974 were with normoglycemia. The follow-up durations were 12 to 168 months (mean: 52.6 months), and 2100 patients with AMI developed MACEs during follow-up. Univariate analyses were used in four studies when the association between prediabetes and MACEs was evaluated [15, 16, 19, 20], while multivariate analyses were used in the other eight studies [14, 15, 18, 19, 22, 28–30]. The NOS of the included studies were six to nine stars, suggesting overall moderate to good study quality (Table 2).

**Table 1:** Study characteristics

Study	Location	Diagnosis	Sample size	Mean age (years)	Men (%)	Definition of PreD	Timing for diagnosis of PreD	No. of patients with PreD	Follow-up duration (months)	No. of patients with MACE	Variables adjusted
Høfsten 2009	Denmark	AMI	128	64.8	71.2	IFG/IGT	At discharge	56	21	39	Age, sex, histories of CHF, LVEF, Killip class, and NT-proBNP
Janszky 2009	Sweden	Nonfatal AMI	938	59.4	70.9	IFG	3 months after AMI onset	251	96	370	Age, sex, obesity, HTN, physical activity, TC, TG, Q wave infarction and education
Knudsen 2011	Norway	STEMI	200	57.1	81.5	IFG/IGT	After an overnight fast of admission	81	33	52	None
Donahue 2011	USA	First AMI	986	55	73.7	IFG	4.4 months after AMI onset	390	54	195	Age, sex, current smoker, alcohol drinking, HTN, dyslipidemia, aspirin use, and BMI
Mazurek 2012	Poland	Invasively treated AMI	1718	59	77.4	IFG/IGT	At discharge	936	38	689	None
Tamita 2012	Japan	AMI	190	61.9	78	IFG/IGT	At discharge	112	64	59	Age, sex, HbA1c, FPG, admission PG, previous HTN, stroke, MI, and CABG, diuretics and statins use
Ritsinger 2015	Sweden	AMI	112	63.2	71.3	IGT	At discharge	58	139	46	None
Belenkova 2015	Russia	STEMI	461	60.8	69.6	IGT	At discharge	32	12	103	None
Pararajasingam 2019	Denmark	First AMI	155	60	84.5	IFG/IGT	Within 3 days after admission	70	168	58	Age, sex, and type of AMI
Sardu 2019	Italy	AMI	360	67.6	54.2	IFG, IGT or HbA1c (5.7–6.4%)	NR	180	12	23	Age, sex, BMI, SBP, DBP, HR, CV risk factors, TC, LDL-C, Scr, and concurrent medications
Karayiannides 2021	Sweden	AMI	781	63.7	75.9	IFG, IGT or HbA1c (5.7–6.4%)	NR	461	58	344	Age and sex
Gao 2021	China	MINOCA	943	54.9	75.8	HbA1c (5.7–6.4%)	NR	371	42	122	Age, sex, BMI, AMI type, HTN, and dyslipidemia

PreD, prediabetes; MACE, major adverse cardiovascular events; AMI, acute myocardial infarction; STEMI, ST segment elevation myocardial infarction; MINOCA, myocardial infarction with nonobstructive coronary arteries; IFG, impaired fasting glucose; IGT, impaired glucose tolerance; HbA1c, hemoglobin A1c; NR, not reported; CHF, chronic heart failure; LVEF, left ventricular ejection fraction; NT-proBNP, N-terminal pro B-type natriuretic peptide; HTN, hypertension; TC, total cholesterol; TG, total glyceride; LDL-C, low-density lipoprotein cholesterol; BMI, body mass index; SBP, systolic blood pressure; DBP, diastolic blood pressure; HR, heart rate; CV, cardiovascular; Scr, serum creatinine; PG, plasma glucose; FPG, fasting plasma glucose; MI, myocardial infarction; CABG, coronary artery bypass graft

**Table 2:** Study quality assessment via the Newcastle-Ottawa Scale

Study	Representativeness of the exposed cohort	Selection of the non-exposed cohort	Ascertainment of exposure	Outcome not present at baseline	Control for age and sex	Control for other confounding factors	Assessment of outcome	Enough long follow-up duration	Adequacy of follow-up of cohorts	Total
Høfsten 2009	1	1	1	1	1	1	1	0	1	8
Janszky 2009	1	1	1	1	1	1	1	1	1	9
Knudsen 2011	1	1	1	1	0	0	1	0	1	6
Donahue 2011	1	1	1	1	1	1	1	1	1	9
Mazurek 2012	1	1	1	1	0	0	1	1	1	7
Tamita 2012	1	1	1	1	1	1	1	1	1	9
Ritsinger 2015	1	1	1	1	0	0	1	1	1	7
Belenkova 2015	1	1	1	1	0	0	1	0	1	6
Pararajasingam 2019	1	1	1	1	1	0	1	1	1	8
Sardu 2019	1	1	1	1	1	1	1	0	1	8
Karayiannides 2021	1	1	1	1	1	0	1	1	1	8
Gao 2021	1	1	1	1	1	1	1	1	1	9

### Results of the meta-analysis

**Fig. 2:**
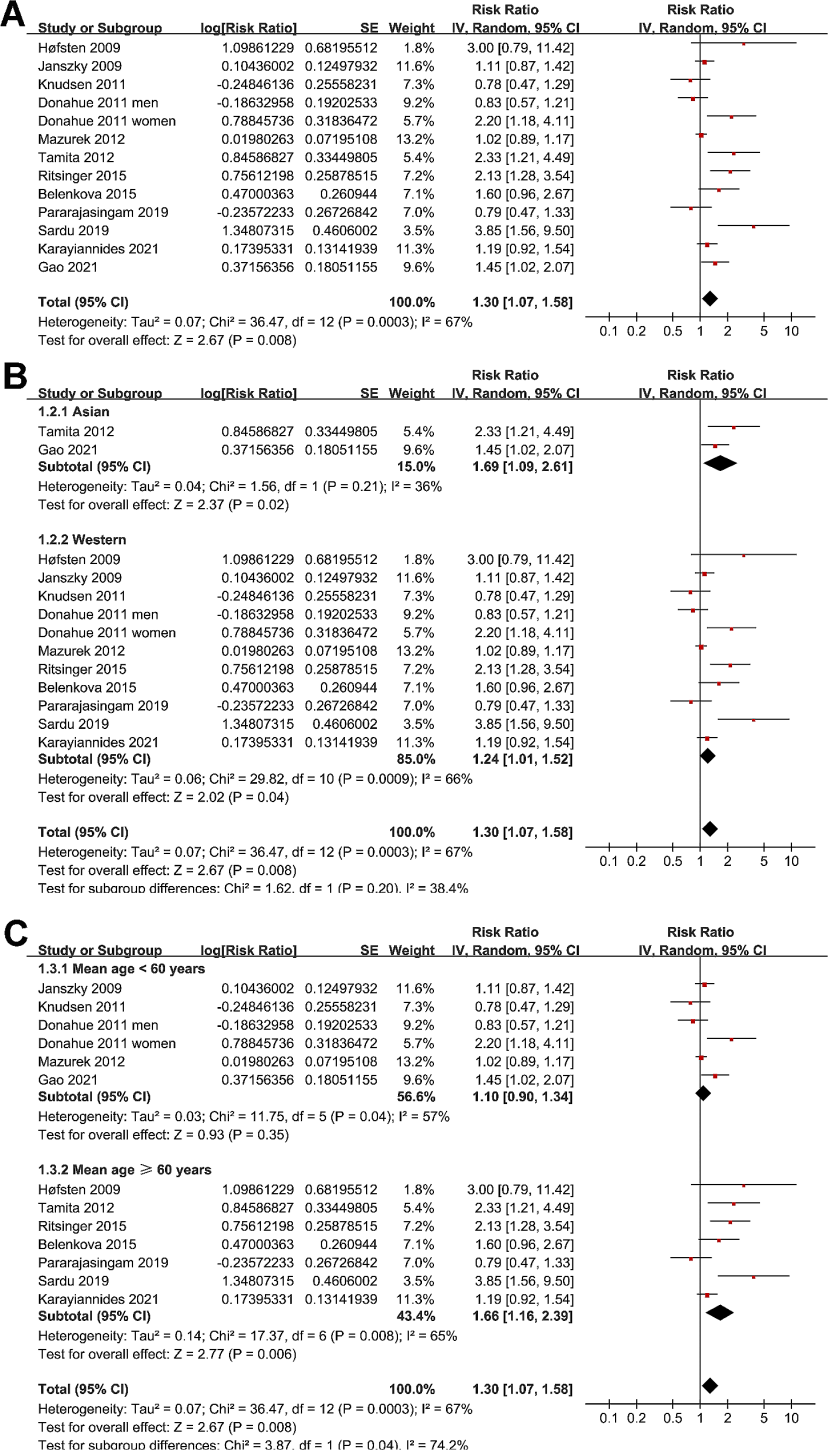
Forest plots for the meta-analysis of the association between prediabetes and long-term risk of MACEs after AMI; (**A**), overall meta-analysis; (**B**), subgroup analysis according to study country; and (**C**), subgroup analysis according to age of the patients

One study reported the outcomes in men and women separately [13], and accordingly, these two datasets were independently included in the meta-analysis. Data of RR were reported in four studies [15, 16, 19, 20], and data of HR were reported in eight studies [14, 15, 18, 19, 22, 28–30]. The pooled results showed that compared to those with normoglycemia, AMI patients with prediabetes were associated with a higher incidence of MACEs during follow-up (RR: 1.30, 95% CI: 1.07 to 1.58, *p* = 0.008; I^2^ = 67%; Fig. 2A). A subgroup analysis did not support that the results were significantly different between studies reporting RR and HR (p for subgroup difference = 0.71; Supplemental Fig. 1).

**Fig. 3:**
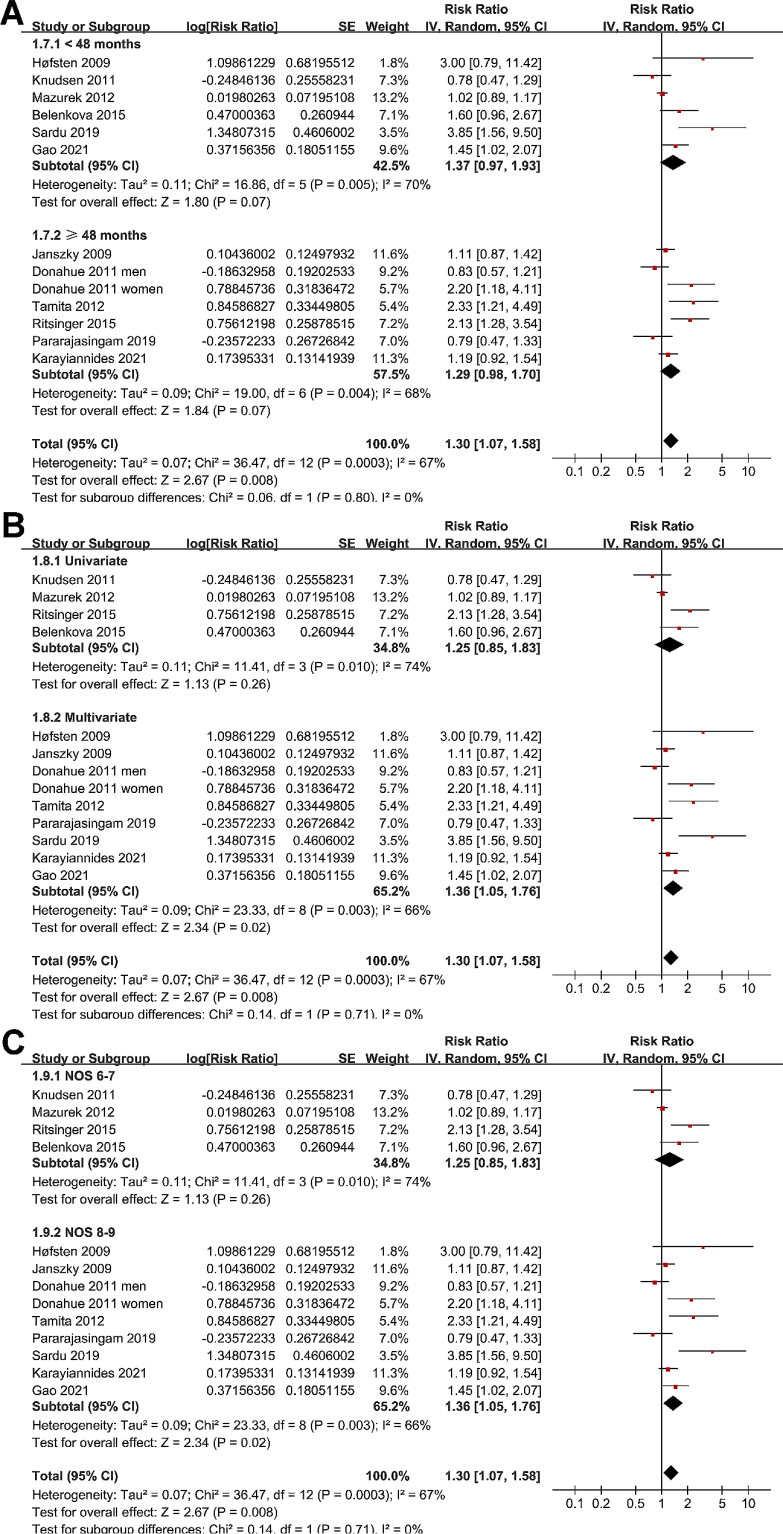
Forest plots for the subgroup analyses of the association between prediabetes and long-term risk of MACEs after AMI; (**A**), subgroup analysis according to proportion of men; (**B**), subgroup analysis according to the definitions of prediabetes; and (**C**), subgroup analysis according to the timing for the elevation of prediabetes

**Fig. 4:**
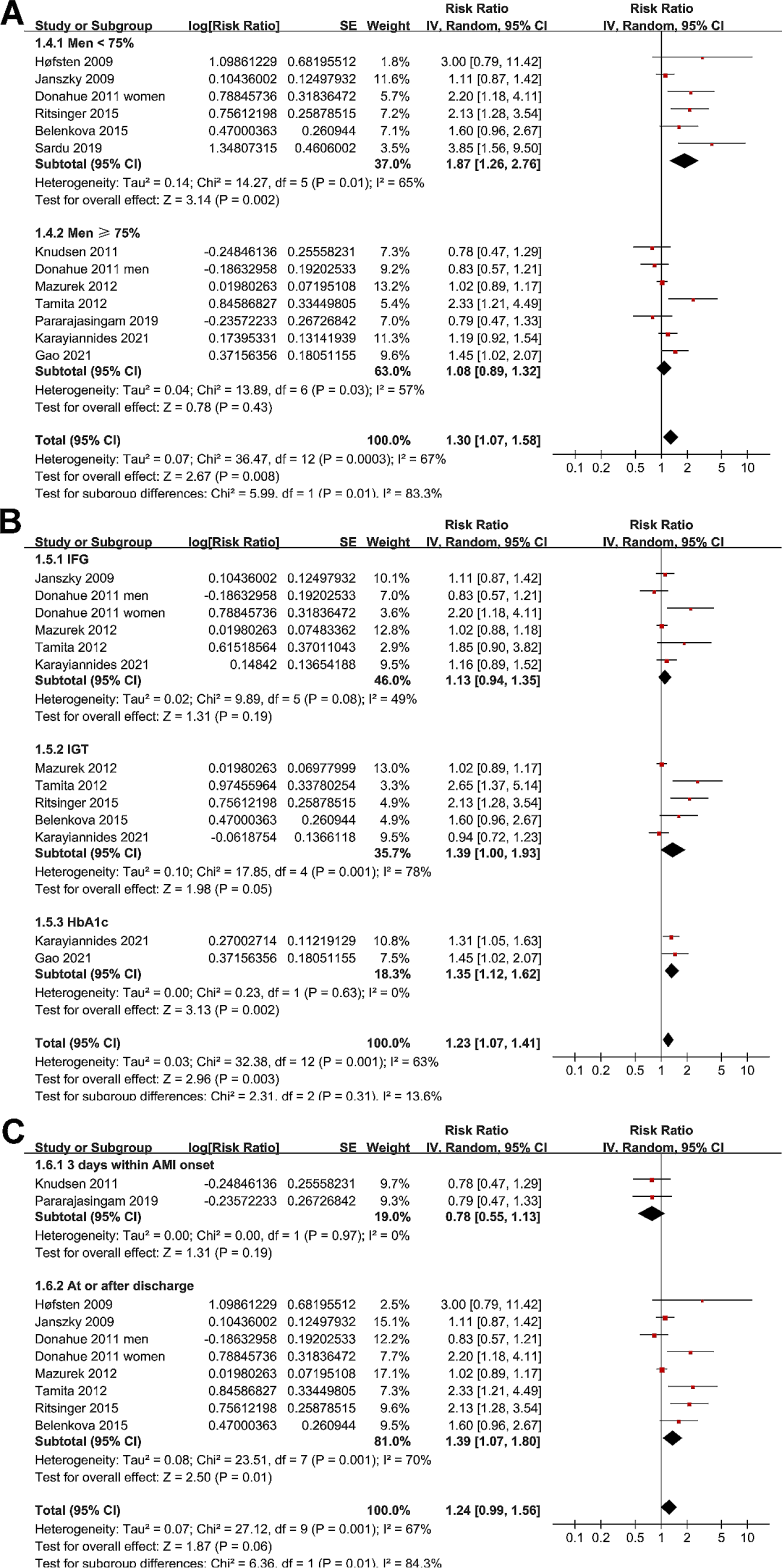
Forest plots for the subgroup analyses of the association between prediabetes and long-term risk of MACEs after AMI; (**A**), subgroup analysis according to follow-up durations; (**B**), subgroup analysis according to the analytic models (univariate or multivariate); and (**C**), subgroup analysis according to the study quality scores

Sensitivity analyses by excluding one dataset at a time (leave-one-out test) did not significantly affect the results (RR: 1.24 to 1.36, p all < 0.05). Subsequent subgroup analysis showed similar results in studies from Asian and non-Asian countries (p for subgroup difference = 0.20; Fig. 2B), while a stronger association between prediabetes and MACEs after AMI was observed in studies of patients with mean age ≥ 60 years compared to < 60 years (RR: 1.66 versus 1.10, p for subgroup difference = 0.04; Fig. 2C), and in studies with proportion of men < 75% compared to ≥ 75% (RR: 1.87 versus 1.08, p for subgroup difference = 0.01; Fig. 3A). Subgroup analyses did not support that different definition of prediabetes (IFG, IGT, or mildly elevated HbA1c) could significantly affect the association between prediabetes and the risk of MACEs (p for subgroup difference = 0.31; Fig. 3B). However, prediabetes was shown to be associated with the risk of MACEs in studies with prediabetes evaluated at or after discharge, but not in studies with prediabetes evaluated within three days of AMI onset (RR: 1.39 versus 0.78, p for subgroup difference = 0.01; Fig. 3C). Further subgroup analyses according to follow-up duration (p for subgroup difference = 0.80; Fig. 4A), analytic models (p for subgroup difference = 0.71; Fig. 4B), or study quality scores (p for subgroup difference = 0.71; Fig. 4C) did not significantly affect the results.

**Fig. 5:**
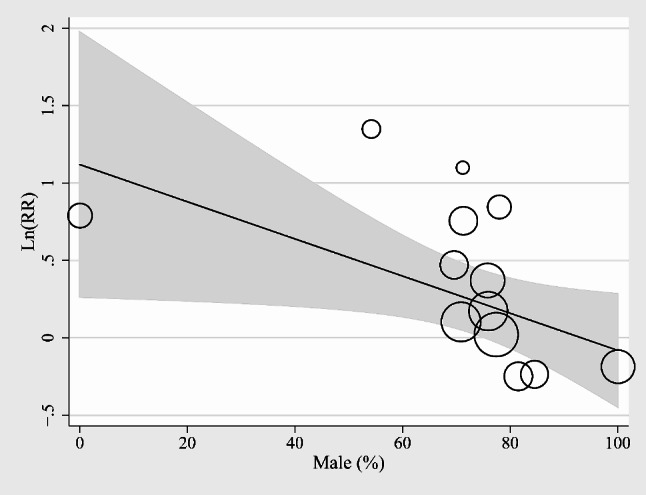
Univariate meta-regression analyses showed that proportion of men in each study is negatively correlated to the association between prediabetes and long-term incidence of MACEs after AMI

Finally, the results of the meta-regression analysis suggested that the proportion of the males in each study was negatively correlated with the association between prediabetes and the incidence of MACEs during follow-up (coefficient = -0.012, *p* = 0.03; Table 3; Fig. 5), while other factors such as sample size, mean age, follow-up duration, or NOS did not seem to significantly modify the results.

**Table 3:** Results of univariate meta-regression analysis

	RR for the association between prediabetes and MACE		
Variables	Coefficient	95% CI	P values
Sample size	-0.00031	-0.00087 to -0.00024	0.24
Mean age (years)	0.065	-0.004 to 0.134	0.07
Men (%)	-0.012	-0.023 to − 0.001	0.03
Follow-up duration (months)	-0.0021	-0.0087 to 0.0045	0.50
NOS	0.053	-0.214 to 0.320	0.67

RR, risk ratio; MACE, major adverse cardiovascular events; CI, confidence interval; NOS, Newcastle-Ottawa Scale

### Publication bias evaluation

**Fig. 6:**
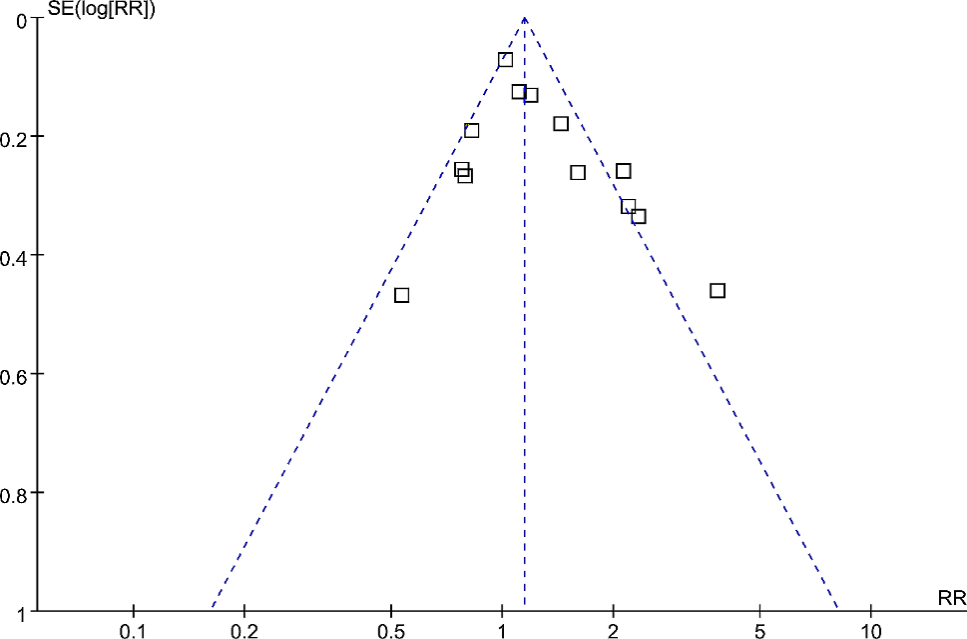
Funnel plots for the meta-analysis of the association between prediabetes and long-term risk of MACEs after AMI

The funnel plots for the meta-analysis of the association between prediabetes and long-term risk of MACEs after AMI are symmetrical on visual inspection, indicating a low risk of publication bias (Fig. 6). Results of Egger’s regression test (*p* = 0.19) also suggested a low risk of publication bias.

## Discussion

The findings of this meta-analysis underscore the significant association between prediabetes and an elevated long-term risk of MACEs in patients following AMI. By synthesizing data from twelve prospective cohort studies encompassing a total of 6972 individuals with AMI, our analysis revealed a 30% higher incidence of MACEs among those with prediabetes compared to their normoglycemic counterparts during a mean follow-up period of 52.6 months. These findings highlight the important influence of glycemic dysregulation on the prognosis of patients with AM, even before the diagnosis of diabetes.

The observed association between prediabetes and an elevated risk of MACEs following AMI prompts a deeper exploration into the underlying mechanistic links driving this relationship. One of the key mechanisms through which prediabetes may predispose individuals to MACEs following AMI is the exacerbation of atherosclerotic plaque vulnerability [31]. Insulin resistance, a hallmark of prediabetes, fosters a pro-inflammatory and pro-thrombotic milieu within the vasculature, promoting endothelial dysfunction, oxidative stress, and dyslipidemia [32]. These perturbations contribute to the formation of unstable atherosclerotic plaques characterized by increased lipid deposition, inflammatory cell infiltration, and propensity for rupture or erosion, thereby augmenting the risk of acute coronary events such as AMI [33]. Furthermore, prediabetes exerts deleterious effects on myocardial structure and function, exacerbating the myocardial injury incurred during AMI and predisposing to adverse cardiac remodeling [34]. Insulin resistance-mediated activation of the renin-angiotensin-aldosterone system, sympathetic nervous system hyperactivity, and myocardial fibrosis contributes to myocardial hypertrophy, interstitial fibrosis, and diastolic dysfunction, rendering the myocardium more vulnerable to ischemic injury and adverse remodeling following AMI [35]. Additionally, dysregulated glucose metabolism impairs myocardial energetics and substrate utilization, exacerbating myocardial ischemia-reperfusion injury and impairing post-infarction recovery [36]. Finally, prediabetes fosters a systemic pro-inflammatory state [37]. Chronic low-grade inflammation promotes endothelial dysfunction, thrombogenesis, and plaque instability, thereby exacerbating the risk of adverse cardiovascular events post-AMI [38]. The key molecular signaling pathways underlying these potential mechanisms remain to be determined.

Subgroup analyses further elucidated several intriguing observations regarding the nuanced interplay between prediabetes and cardiovascular outcomes in this population. Notably, our findings suggest a stronger correlation between prediabetes and adverse cardiovascular outcomes in studies comprising older patients (mean age ≥ 60 years) and a lower proportion of men (< 75%). These observations hint at potential demographic disparities in the impact of prediabetes on cardiovascular risk following AMI, warranting further investigation into underlying pathophysiological mechanisms and tailored risk management strategies. Moreover, the timing of prediabetes evaluation emerged as a crucial determinant of its prognostic significance in AMI patients. Interestingly, while prediabetes assessed at or after discharge was associated with a heightened risk of MACEs, no such association was evident when prediabetes was evaluated within three days of AMI onset. These findings are consistent with the previous of a previous study which showed that evaluating for glycemic disorder in the acute phase of AMI may not accurately reflect the long-term risk of dysglycemia in these patients [39]. In addition, this temporal discrepancy may also suggest a dynamic interplay between glycemic status and cardiovascular outcomes during different phases of AMI management, possibly influenced by varying degrees of myocardial injury, inflammatory response, and metabolic perturbations. Clinically, these findings underscore the importance of comprehensive glycemic assessment beyond the acute phase of AMI and highlight the potential utility of early identification and intervention in mitigating long-term cardiovascular risk among individuals with prediabetes. Furthermore, our analysis did not reveal significant differences in the association between prediabetes and MACEs based on the specific criteria used to define prediabetes (e.g., IFG, IGT, or mildly elevated HbA1c). This suggests a consistent impact of prediabetes across different diagnostic thresholds, emphasizing the clinical relevance of identifying and addressing even subtle disturbances in glucose metabolism in the context of AMI.

A recent meta-analysis suggested among patients who underwent PCI for coronary artery disease (CAD), the risk of all-cause and cardiac mortality, major adverse cardiovascular events and MI in prediabetic patients was higher compared with normoglycemic patients [40]. However, studies with patients of various subtype of CAD, such as stable CAD, unstable angina (UA), and MI were all included in the meta-analysis, which may lead to heterogeneity [40]. In addition, retrospective cohort studies were also included in the previous meta-analysis, which may introduce additional recall and selection biases [40]. Moreover, the results were based on data from univariate analysis, and the influences of potential confounding factors could not be determined [40]. Our meta-analysis has several methodological strengths compared to the previous one, such as extensive literature search, focusing solely on patients with MI, including only prospective studies to minimize the influence of recall and selection bias, and performing multiple sensitivity, subgroup, and meta-regression analyses to validate the robustness of the findings. While our meta-analysis provides valuable insights into the prognostic implications of prediabetes in AMI patients, several limitations warrant consideration. The protocol of the meta-analysis was not prospectively registered in PROSPERO. Differences in variables adjusted among each study may potentially affect the results and contribute to heterogeneity. However, a subgroup analysis showed similar results in studies with univariate and multivariate analyses. In addition, the influences of main treatments for AMI on the results could not be determined because the stratified data based on treatments of AMI were largely not reported among these studies, which warranted further investigation in future studies. As a meta-analysis of observational studies, we could not determine if the association between prediabetes and an increased long-term risk of MACEs after AMI is causative. Future studies are still needed to determine the optimal evaluation and management protocol of AMI patients with prediabetes.

## Conclusions

In conclusion, this meta-analysis underscores the heightened risk of MACEs associated with prediabetes in patients following AMI and sheds light on potential modifiers of this association, including demographic factors and timing of prediabetes evaluation. These findings underscore the importance of comprehensive glycemic assessment and targeted risk management strategies in optimizing cardiovascular outcomes among individuals with prediabetes recovering from AMI. Moving forward, further research is warranted to elucidate the underlying mechanisms driving this association and to evaluate the efficacy of tailored interventions aimed at attenuating cardiovascular risk in this high-risk population.

### Electronic supplementary material

Below is the link to the electronic supplementary material.

Supplementary Material 1

## Data Availability

All data generated or analyzed during this study are included in this published article.
