# Supplementary figures and images for: Influence of prediabetes on the prognosis of patients with myocardial infarction: a meta-analysis
Source: Diabetol Metab Syndr. 2024 Jul 12;16:160. doi: 10.1186/s13098-024-01381-1 (PMC11241782; doi:10.1186/s13098-024-01381-1)

**Supplemental Figure 1:** Subgroup analysis in studies reporting RR and HR


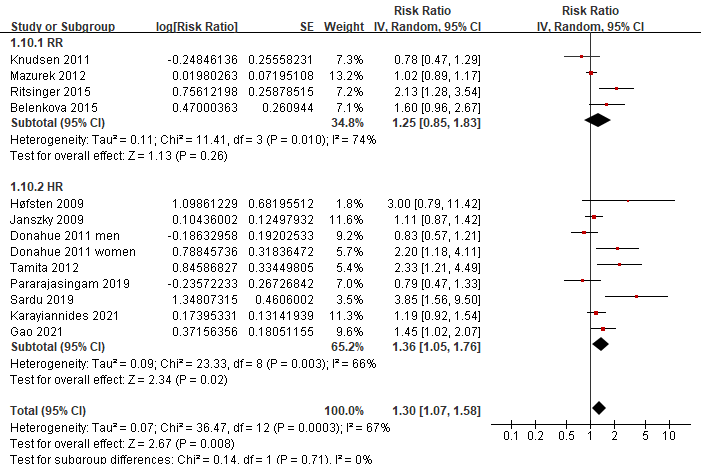

Supplement: Supplementary file 1 — Supplementary Material 1 [file 13098_2024_1381_MOESM1_ESM.docx]
